# Reverse Semi‐Combustion Driven by Titanium Dioxide‐Ionic Liquid Hybrid Photocatalyst

**DOI:** 10.1002/cssc.202001717

**Published:** 2020-09-07

**Authors:** Muhammad I. Qadir, Marcileia Zanatta, Jose Pinto, Isabel Vicente, Aitor Gual, Emily F. Smith, Brenno A. D. Neto, Paulo E. N. de Souza, Sherdil Khan, Jairton Dupont, Jesum Alves Fernandes

**Affiliations:** ^1^ Institute of Chemistry Federal University of Rio Grande do Sul Campus Agronomia Porto Alegre 90650-001 Brazil; ^2^ Department of Nanocatalysis J. Heyrovský Institute of Physical Chemistry, Czech Academy of Sciences Dolejškova 2155/3 18223 Prague 8 Czech Republic; ^3^ i3N|Cenimat, Department of Materials Science NOVA School of Science and Technology NOVA University Lisbon 2829-516 Caparica Portugal; ^4^ School of Chemistry University of Nottingham University Park Nottingham NG7 2RD United Kingdom; ^5^ Unitat de Tecnologíe Químiques EURECAT Tarragona 43007 Spain; ^6^ Institute of chemistry University of Brasília Campus Universitário Darcy Ribeiro Brasília 70904-970 Brazil; ^7^ Institute of Physics University of Brasília Campus Universitário Darcy Ribeiro Brasília 70904-970 Brazil; ^8^ Institute of Physics Federal University of Rio Grande do Sul Campus Agronomia Porto Alegre 90650-001 Brazil

**Keywords:** carbon dioxide, carbon monoxide, ionic liquids, photocatalysis, titania

## Abstract

Unprecedented metal‐free photocatalytic CO_2_ conversion to CO (up to 228±48 μmol g^−1^ h^−1^) was displayed by TiO_2_@IL hybrid photocatalysts prepared by simple impregnation of commercially available P25‐titanium dioxide with imidazolium‐based ionic liquids (ILs). The high activity of TiO_2_@IL hybrid photocatalysts was mainly associated to (i) TiO_2_@IL red shift compared to the pure TiO_2_ absorption, and thus a modification of the TiO_2_ surface electronic structure; (ii) TiO_2_ with IL bearing imidazolate anions lowered the CO_2_ activation energy barrier. The reaction mechanism was postulated to occur via CO_2_ photoreduction to formate species by the imidazole/imidazole radical redox pair, yielding CO and water.

## Graphical abstract

**Hybrid theory**: An effective method to prepare TiO_2_@IL semiconductor‐liquid junctions for CO_2_ photo‐conversion is developed. The ionic liquid (IL) plays a dual role by inducing a shift of the valence band with simultaneously decreasing the band gap as well as continuous generation of formate radicals, which suggests a decrease of CO_2_ activation energy barrier and thus improves the catalyst performance (yield and selectivity to CO).

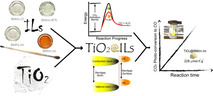

## Introduction

During the last decade, many research efforts have been focussed on the development of efficient catalytic processes involving the use of renewable energy sources for the carbon neutral transformation of carbon dioxide (CO_2_) to fuels and chemicals.^1^ Among them, CO_2_ photoreduction to intermediates for chemical synthesis [carbon monoxide (CO)] and/or solar fuels for storage/transport of energy in the form of hydrocarbons (artificial photosynthesis) has grown into a blooming field of research.^2^ A simple combination of sunlight, aqueous solutions saturated with CO_2_ and an appropriate photocatalysts may yield CO (reverse semi‐combustion) and/or solar fuel hydrocarbons (reverse combustion).^3^ However, the high energy barrier of CO_2_ activation, side reactions (such as hydrogen evolution) and high rates of electron‐hole pair recombination of the photocatalysts employed still remain as unsolved challenges.^4^ Although great advances have been made to enhance the CO_2_ photoreduction, the photocatalytic performance (in terms of activity and/or selectivity) reported so far still remain low compared to conventional CO_2_ reduction processes (i. e., CO_2_ thermal reduction and CO_2_ electroreduction).2b Most of the current CO_2_ photoreduction approaches encompass only one side of the reaction by (i) designing new photocatalysts to extend the visible absorption and supress the electron‐hole recombination (ii) or aiming to overcome the formation of the undesired high‐energy intermediate species.^5^ Therefore, a fresh approach that embraces both sides of the reaction (semiconductor properties and CO_2_ activation) should be sought to unlock the next generation of photocatalysts for competitive and efficient CO_2_ photoreduction by innovative approach exploiting fleeting open‐shell intermediates, such as radical ions and radicals.^6^

Titanium dioxide (TiO_2_) has been widely studied as a photocatalyst in the production of solar fuels,^7^ however, still displaying low photocatalytic activity for the CO_2_ conversion, either, to CO and solar fuel hydrocarbons (Table S2).^8^ Among others, the most utilised strategy for the enhancement of the TiO_2_ photocatalytic performance, involve the structural and surface fine tuning by incorporation of other semiconductors (i. e., Z‐schemes),^9^ dopants,^10^ metal‐nanoparticles,^11^ thus shifting the absorption edge to the visible light.3a

The pH of the reaction media, and thus, the concentration in solution by the formation of carbonate ([CO_3_]^2−^) and bicarbonate ([HCO_3_^−^]) species is also an important factor for enhancing the CO_2_‐tranformation reaction rate kinetics. For instance, the superior CO_2_‐transformation performance of TiO_2_ anatase (001) surface is explained by a stronger basicity of the surface oxygen sites.^12^ Most importantly, mass transfer limitations plays a crucial role, since compared to the gas‐solid interface, the energy barrier for the liquid‐solid CO_2_‐photocatalysed reduction is reduced by 0.05–0.25 eV in aqueous saturated CO_2_ solutions with high bicarbonate ([HCO_3_^−^]) concentrations. The water solvation effect can greatly decrease the energy barrier of CO_2_ reduction and also affect the selectivity of the reaction processes by providing water dissociation species involved in most of the common CO_2_‐reduction equations (i.e., protons ([H^+^]) or hydroxy species ([OH^−^]).^13^

Imidazolium‐based ionic liquids (ILs) have been recognized to solubilize and activate CO_2_ by stabilizing radical/anionic species^14^ and hence, may constitute an attractive material for CO_2_ photoreduction.^15^

**Figure 1:**
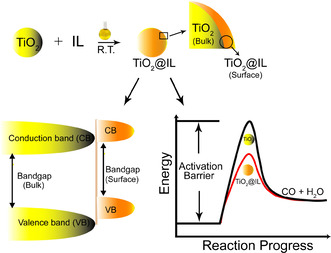
Schematic representation of expected effects induced by ILs on TiO_2_ surface for photoreduction of CO_2_ in aqueous solution. a) TiO_2_@IL hybrid photocatalyst preparation. b) TiO_2_@IL surface valence and conduction band edge shift with simultaneously decreasing the band gap when compared to bare TiO_2_. c) TiO_2_@IL lowering the activation barrier energy of CO_2_ photoreduction, similar to proposed for electroreduction of CO_2_ to CO by Masel and co‐workers.14a

Therefore, combining ILs with semiconductors is a promising strategy towards the modification of physical‐chemical properties of the semiconductors (Figure 1).^16^

Recently, many studies have been focussing on the interaction of ILs and semiconductors.^17^ Based on elegant theoretical calculations, the interactions of ILs with TiO_2_ have been proposed to affect and modulate the electronic properties of the TiO_2_ surface (i. e., valence and conduction band edge energies and modification of the band gap).^18^ In this case, the IL may provide the driving force towards photocatalytic redox process by formation of a solid semiconductor‐liquid junction improving the charge separation and tuning the electron/hole ratio. Moreover, aqueous solution of ILs containing basic anions when in contact with CO_2_ can favour its solubilisation by bicarbonates ([HCO_3_^−^]) concentrations^19^ and decrease the energy barrier of CO_2_ reduction.14a, 20

Herein, we demonstrate that the simple impregnation of commercially available P25‐TiO_2_ with imidazolium ILs generates highly efficient hybrid photocatalysts for the CO_2_ reduction to CO with unprecedented catalytic activity. We demonstrate that the enhancement of the CO_2_‐photocalytic performance is associated to (i) a shift of the TiO_2_@IL valence and conduction band edge with simultaneously decreasing the band gap when compared to bare TiO_2_; (ii) continuous generation of formate species via imidazole/imidazole radical redox pair lowered the activation barrier energy of CO_2_ photoreduction (Figure 1).

## Results and Discussion

The photoreduction of CO_2_ was first performed in aqueous solution of ILs,^21^ without the presence of TiO_2_ (Table 1). The ILs 1‐*n‐*butyl‐3‐methylimidazolium bis(trifluoromethylsulfonyl)imide ([BMIm][NTf_2_]) and 1‐*n‐*butyl‐3‐methylimidazolium chloride ([BMIm]Cl) ILs did not show production of CO (Table 1, entries 1 and 2). In contrast to ILs with [NTf_2_^−^] and Cl^−^ anions, ILs with imidazolate anion ([Im]) 1‐*n‐*butyl‐3‐methylimidazolium imidazolate ([BMIm][Im]) displayed CO_2_ photoreduction to CO up to 62±30 μmol g^−1^ with selectivity higher than 99 % (Table 1, entry 3).

**Table 1:** Summary of photoreduction performance for CO_2_ reduction using IL in aqueous media.^[a]^

Entry	Catalyst	IL [mg]	CO [μmol g^−1^]
1	[BMIm]Cl	0.8	–
2	[BMIm][NTf_2_]	0.8	–
3	[BMIm][Im]	0.8	62±30
4	[BMPy][Im]	0.8	60±32
5	[(But)_3_EP][Im]	0.8	89±38

Reaction conditions: [a] CO_2_ (atmospheric pressure), H_2_O (2 mL), 25 °C, Xe lamp (300 W), 2 h. CO production values have been obtained from at least three experiments.

There is a noteworthy influence of the anion on CO_2_ photoreduction (Table 1, entries 1–5). CO is produced only with ILs containing basic anions (imidazolate), indicating their involvement (direct or indirect) on the CO_2_ activation, H_2_O oxidation, and probably also on the confinement of the photocatalyst active sites in water‐deficient media, in which the CO_2_ reduction mechanism follows distinct reaction pathways than in H_2_O‐rich media (i. e., higher H_2_ selectivity in H_2_O‐rich media). It is important to note that on replacing the imidazolium cation with pyrrolidinium and phosphonium cation, ILs also produced a significant amount of CO, approximately 60±32 and 89±38 μmol g^−1^ with [BMPy][Im] and [(But)_3_EP][Im] ILs, respectively (Table 1, entries 4 and 5). This can be related to the presence of imidazolate anion not only increasing the sorption of CO_2_ but also increasing the local basicity. The photoreduction of ^13^CO_2_ with the aqueous solution of [BMIm][Im] was performed to verify that CO is produced from the CO_2_ by photoreduction leading to ^13^CO detected at *m/z*=29 by GC‐MS (Figure S11). Of note, no CO was observed when the reactions were performed under dark using ILs.

TiO_2_@IL hybrid photocatalysts were prepared by simple mixing of P25‐TiO_2_ (AEROXIDE TiO_2_ P25)^22^ with [BMIm][Im], [BMIm][NTf_2_], [BMIm]Cl, [BMPy][Im] and [(But)_3_EP][Im] ILs at room temperature, in water or chloroform yielding a similar IL loading for all of them (see the Supporting Information for details). Brunauer‐Emmett‐Teller (BET) analyses show that both the TiO_2_ surface area and pore volume are significantly reduced after IL impregnation (Table S1 and Figure S5b). These results indicate that the ILs are well distributed among TiO_2_ grains in addition to uniformly filling the apparent porosity created by interstitial space between TiO_2_ grains.

It is important to mention that IL (cations and anions) coordination on TiO_2_ surface is still not fully understood; however, it has been accepted that cations and anions coordinate to different TiO_2_ sites.17b, 18, 23 The TiO_2_ surface offers either O or Ti atoms for ionic coordination; positively charged cations coordinate to the O atom and negatively charged anions coordinate to Ti atoms.^18^ For TiO_2_, the valence band predominantly consists of O 2p states, and the conduction band is mainly composed of Ti 3d states.^24^ Therefore, TiO_2_ band edge shift depends on the amount of electronic charge accepted/donated from the IL to TiO_2_ or vice versa.^18^ In order to investigate these effects UV/Vis and X‐ray photoelectron spectroscopy (XPS) measurements of TiO_2_@IL hybrid photocatalysts were performed.

**Figure 2:**
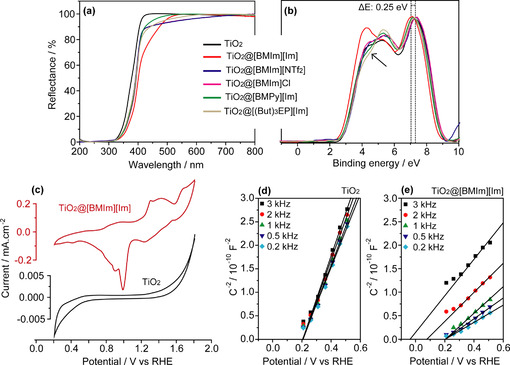
(a) Diffuse reflectance spectra and (b) valence band spectra from XPS. Electrochemistry measurements of pure TiO_2_ versus TiO_2_@[BMIm][Im]: (c) Cyclic voltammetry and (d, e) Mott‐Schottky plots.

UV/Vis measurements of TiO_2_@IL revealed a red shift (≈0.2 eV) compared with bare TiO_2_, regardless of the type of IL used (Figure 2a). These results are in agreement with theoretical calculations on TiO_2_@IL interaction, in which a red shift between 0.1 to 0.4 eV is predicted.^18^ XPS wide‐scan and high‐resolution spectra of C 1 s and Ti 2p from all TiO_2_@IL can be found in the Supporting Information (Figures S7 and S8). From the valence band XPS analyses (Figure 2b), the valence band maximum (VBM) of bare TiO_2_ surface was observed at approximately 5 eV ascribed to O 2p–Ti 4sp *π* bonding and the higher binding energy state was observed at approximately 7.5 eV that is assigned to O 2p–Ti 3d *σ* bonding.^24^, ^25^ On the other hand, a new structure at TiO_2_ surface is observed for TiO_2_@[BMIm][Im] by a shift of the VBM (0.25 eV) towards the Fermi energy *E*_f_ . The shift of VBM at lower energy in the TiO_2_ system can be ascribed to the presence of new surface states within the TiO_2_ and IL interface (a control experiment is displayed in the Figure S9).

The TiO_2_@[BMIm][Im] upward shift of VBM of 0.25 eV led to an upward shift of the conduction band maximum (CBM) of 0.05 eV. The upward shift of VB and CB suggests a higher amount of charge transfer between IL anion and TiO_2_ surface compared to IL cation withdrawing charge from TiO_2_ surface. However, for TiO_2_@[BMPy][Im] and TiO_2_@[(But)_3_EP][Im] a positive shoulder was detected (Figure 2b), which may suggest a downward shift of VBM and thus a higher cation acceptance charge from TiO_2_ surface compared to IL anion and TiO_2_ surface charge transfer. In comparison, for non‐basic IL anions, TiO_2_@[BMIm][NTf_2_] and TiO_2_@[BMIm]Cl, the VBM position remained as of bare TiO_2_. Therefore, the decrease of 0.2 eV in the BG, observed in the UV/Vis measurements, suggest a downward shift of 0.2 eV in the CBM for TiO_2_@[BMIm][NTf_2_] and TiO_2_@[BMIm]Cl. This effect can be ascribed to a weak interaction between anion and TiO_2_, thus leading to a higher charge donation from TiO_2_ surface to IL cation.^18^ These results confirm that in most of the cases, anions have a dominant influence on energy band shift; however, it also demonstrated that the proper choice of both IL cation and anion can adjust the desired semiconductor physical‐chemical properties.

To further investigate the effects observed by UV/Vis and XPS, electrochemistry measurements of TiO_2_ and TiO_2_@[BMIm][Im] films deposited on fluorine‐doped tin oxide (FTO) substrate were performed. A significant difference was recorded in cyclic voltammetry of TiO_2_@[BMIm][Im] as compared to bare TiO_2_ (Figures S2c and S10). In line with the literature, bare TiO_2_ did not exhibit peaks in the voltammograms whereas TiO_2_@[BMIm][Im] presented distinct redox peaks and an enhancement in current density.^26^ The appearance of these peaks suggests generation of oxygen vacancies leading to interband states induced by the IL in TiO_2_ crystal structure, thereby resulting in higher conductivity of TiO_2_@[BMIm][Im].^27^ This effect has been previously observed, which was associated to the interfacial electric field effect and/or to the water contaminants in ILs.^28^ The flat band positions of bare TiO_2_ and TiO_2_@[BMIm][Im] were studied through Mott‐Schottky (MS) plots (Figure 2d,e). MS plots display positive slopes corresponding to the n‐type nature of the samples, however, the slope for TiO_2_@[BMI][Im] is smaller than the bare TiO_2_ indicating increased donor densities corroborating to the appearance of redox peaks in cyclic voltammograms (Figure 2c).^29^ For bare TiO_2_ the MS curves converge to approximately 0.2 V vs. reversible hydrogen electrode (RHE) at all applied frequencies. However, TiO_2_@[BMIm][Im] displays frequency dispersion where at lower frequencies the MS curves converge to approximately 0.2 V vs. RHE and for higher frequencies a negative shift is observed. The validity of MS analyses is based on the fact that series capacitances corresponding to the semiconductor‐liquid interfaces are much higher than the capacitance of space charge layer. In case of thin space charge layer (higher defect densities in electrode material), the capacitance such as Helmholtz capacitance may not be neglected, which may result in frequency dispersion.^30^ It should be noted that the dispersion in MS plots is a topic of discussion; however, possible reasons to this dispersion are defective nature of the film, interband states, inhomogeneous doping and atomic roughness.^30^, ^31^ Therefore, electrochemical, XPS and UV/Vis analyses clearly demonstrated a shift of valence band position and generation of interband states in TiO_2_ surface due to impregnation of [BMIm][Im] IL.^32^

The photocatalytic experiments of the thus prepared TiO_2_@IL hybrid photocatalysts were also performed using only water. The obtained results are summarized in Table 2.

**Table 2:** Summary of photoreduction performance for CO_2_ reduction using TiO_2_@IL hybrid photocatalyst in aqueous media.^[a]^

Entry	Catalyst	TiO_2_@IL [mg]	CO [μmol g^−1^]
1	TiO_2_@[BMIm][Im]	20^[b]^	455±96
2	TiO_2_@[BMPy][Im]	20^[b]^	80±7
3	TiO_2_@[(But)_3_EP][Im]	20^[b]^	207±16
4	TiO_2_@[BMIm]Cl	20^[b]^	220±23
5	TiO_2_@[BMIm][NTf_2_]	20^[b]^	101±55
6	TiO_2_+[BMIm][Im]	20^[b,c]^	177±84
7	TiO_2_	–	3±1

Reaction conditions: [a] CO_2_ (atmospheric pressure), H_2_O (2 mL), 25 °C, Xe lamp (300 W), 2 h. CO production values have been obtained from at least three experiments. [b] The amount of IL used for TiO_2_@IL can be found in Table S1. [c] A small amount of oxygen was detected along with other major by‐products formic acid and bicarbonate (Figures S2–S4).

Naked TiO_2_ revealed very low catalytic activity and produced only CO (Table 2, entry 7, 3±1 μmol g^−1^). Remarkably, TiO_2_@[BMIm][Im] hybrid photocatalyst displays generation of CO of 228±48 μmol g^−1^ h^−1^ with a selectivity of the 99 %, and enhancement of approximately 150 times compared to bare TiO_2_ and 8 times of that of the bare IL (Table 2, entry 1 and Table 1, entry 3). Moreover, TiO_2_@[BMIm][Im] displayed an apparent quantum efficiency of 10.9 % (using 360 nm band pass filter) as well as showed high stability after recyclability tests performed for three cycles of 12 h each (Figure S14). These are impressive results compared to previous systems using sacrificial agents and noble metals reported to date (Tables S2 and S3). TiO_2_@[BMIm][Im] catalyst showed the highest activity to CO, as compared to photocatalysts containing the imidazolate anion associate with phosphonium or pyrrolidinium cations (Table 2, entries 2 and 3). These results can be ascribed to higher charge transfer between IL anion and TiO_2_, and thus the VBM shift of TiO_2_@[BMIm][Im] compared to TiO_2_@[BMPy][Im] and TiO_2_@[(But)_3_EP][Im]. The TiO_2_@[BMIm][Im] also displayed higher conversion of CO_2_ to CO when compared to TiO_2_@[BMIm]Cl and TiO_2_@[BMIm][NTf_2_] (Table 2, entries 4 and 5). In this case, it can be related to a weak interaction between those anions (Cl and [NTf_2_] anions) and TiO_2_ surface, and mainly due to the non‐basic of Cl and [NTf_2_] anions.

The CO_2_ reduction step, the base comes from the activation of water by the imidazolate anion to form an imidazole and [HCO_3_^−^] observed by ^13^C NMR spectroscopy (Figure S3).^19^, ^33^ The formation of imidazole radical (Figure S12) can be afford via TiO_2_ charge transfer, which seems to be very efficient in the TiO_2_@IL heterojunction‐like effect, and H^+^ abstraction from the water oxidation. The imidazole radical transfer its charge to CO_2_, then the imidazole is regenerated, and CO_2_ abstracts a proton and electron to generate formate species, as detected in the liquid phase by ^1^H and ^13^C NMR spectroscopy (Figures S4 and S3, respectively).^20^, ^34^ The formate species generates CO and water, which was confirmed via photocatalytic reaction using aqueous solution of formic acid (without the presence of CO_2_), which preferentially yielded CO (Figure S13).

## Conclusion

The simple impregnation of TiO_2_ with imidazolium‐based ionic liquid (IL) associated with basic anions generated a new hybrid highly active photocatalyst for the CO_2_ reduction in water. The observed photocatalytic activity can be related to synergetic effect between ILs and TiO_2_ that follows the order TiO_2_@[BMIm][Im]≫ TiO_2_@[BMIm]Cl> TiO_2_@[(But)_3_EP][Im] ≫TiO_2_@[BMIm][NTf_2_]> TiO_2_@[BMPy][Im]. The IL plays a dual role by inducing a shift of the valence and conduction band edge energies with simultaneously decreasing the band gap; as well as continuous generation of formate radicals ([HCO_2_^.^]) which suggest a decrease of CO_2_ activation energy barrier and thus improves the catalyst performance (i. e., yield and selectivity to CO). The CO_2_ reduction reaction proceeds probably via CO_2_ reaction with imidazole/imidazole radical redox pair, and then it produces formate species yielding CO and water. This promising approach can be extended to a vast range of photoactive materials (e. g., g‐C_3_N_4_) opening a new avenue towards CO_2_ photoreduction and many others photocatalytic systems.

## Experimental Section

**General**: P25‐TiO_2_ (AEROXIDE TiO_2_ P25) was purchased from EVINIK and it was used without any previous treatment. The ILs 1‐*n‐*butyl‐3‐methylimidazolium imidazolate ([BMIm][Im]), 1‐*n*‐butyl‐1‐methylpyrrolidinium imidazolate ([BMPy][Im]) and tri‐*n*‐butyl‐ethyl‐phosphonium imidazolate ([But)_3_EP][Im]) 1‐*n‐*butyl‐3‐methylimidazolium bis(trifluoromethylsulfonyl)imide ([BMIm][NTf_2_]) and 1‐*n‐*butyl‐3‐methylimidazolium chloride ([BMIm]Cl) were prepared according to literature procedures.^35^ All the ILs were dried under vacuum and argon for 2 days prior to use. ESR analyses were achieved in a Bruker spectrometer (Bruker EMXplus, Germany), equipped with an X‐band (9 GHz) high sensitivity cavity (Bruker ER 4119HS, Germany) using frozen samples inside a quartz finger Dewar filled with liquid nitrogen. 400 μL of the aqueous IL solution (0.22 m for each IL) were collected and transferred to a 1 mL de‐capped syringe and frozen in liquid nitrogen. The frozen cylindrical samples were transferred to a quartz finger Dewar (Noxygen, Germany) filled with liquid nitrogen, placed inside the resonator and their electron spin resonance (ESR) spectra were recorded at −196 °C. This procedure ensured identical volumes for all samples, allowing the quantitative comparison among the recorded ESR spectra. The instrumental settings were 2 mW microwave power, 10 G amplitude modulation, 100 kHz modulation frequency, 1000 G sweep width, 3365 G central field and 50s sweep time. The peak‐to‐peak amplitude, that is the difference between the lowest and the highest amplitudes in the first derivative spectrum, was used to detect signal quantification. NMR analyses were performed on a Bruker Avance 400 spectrometer. XPS measurements were performed using a Kratos AXIS Ultra DLD instrument (details can be found in the Supporting Information, section 6). All the electrochemistry measurements were performed using TiO_2_ and TiO_2_@[BMIm][Im] films deposited on FTO substrate, with each sample being prepared and measured three times to ensure the reproducibility of the measurements (details can be found in the Supporting Information, section 7). The generated gases during the photocatalytic reaction were quantified by GC using an Agilent 6820 equipped with a Porapak Q 80–100 Mesh column and argon as carrier gas. The gaseous products were simultaneously analysed with a thermal conductivity detector (TCD) and a flame ionization detector (FID). Aliquots of 100 μL from the gas phase were removed from the head of the photoreactor reactor and injected with a syringe containing a Hamilton sample lock valve. After the photocatalysis, the liquid phase was analysed by NMR spectroscopy. For the evolutions of intermediates by NMR analyses all the reactions were performed with 120 mg of IL under our standard conditions. In order to detect the generated ^13^CO, a MS (QIC 20®‐Hiden Analytical) configured with the ionization of 70 eV was used.

**CO_2_ photoreduction experiments**: Typically, a Schlenk tube containing 30 mL of degassed milli‐Q water was saturated with CO_2_ (50 bar) at a rate of 2 mL min^−1^ at room temperature. The photocatalysis was performed in a homemade quartz reactor equipped with a water‐circulating jacket to maintain the temperature at 25 °C. 2 mL of CO_2_‐saturated water was added in photoreactor containing the desired amount of freshly prepared catalyst under argon atmosphere. After that, CO_2_ was introduced into the reactor by a filled balloon and stirred at room temperature for 30 min. After removing the CO_2_ balloon, the reactor was placed in front of 300 W Xe lamp. After desired time, gaseous products were withdrawn by gas‐tight syringe from the reactor's headspace and analysed by GC.

## Conflict of interest

The authors declare no conflict of interest.

## Supporting information

As a service to our authors and readers, this journal provides supporting information supplied by the authors. Such materials are peer reviewed and may be re‐organized for online delivery, but are not copy‐edited or typeset. Technical support issues arising from supporting information (other than missing files) should be addressed to the authors.

Supplementary
