# Supplementary material for: Reverse Semi‐Combustion Driven by Titanium Dioxide‐Ionic Liquid Hybrid Photocatalyst
Source: ChemSusChem. 2020 Sep 7;13(20):5580–5. doi: 10.1002/cssc.202001717 (PMC7692890; doi:10.1002/cssc.202001717)
Supplement: Supplementary file 1 — Supplementary [file CSSC-13-5580-s001.pdf]

ChemSusChem

Supporting Information

Reverse Semi-Combustion Driven by Titanium Dioxide-Ionic Liquid Hybrid Photocatalyst

Muhammad I. Qadir, Marcileia Zanatta, Jose Pinto, Isabel Vicente, Aitor Gual, Emily F. Smith, Brenno A. D. Neto, Paulo E. N. de Souza, Sherdil Khan, Jairton Dupont,* and Jesum Alves Fernandes*© 2020 The Authors. Published by Wiley-VCH GmbH. This is an open access article under the terms of the Creative Commons Attribution License, which permits use, distribution and reproduction in any medium, provided the original work is properly cited.

1. Preparation of TiO_2@IL catalysts

The TiO_2@[BMIm][Im] (around of 3 wt%) and [BMIm]Cl (around of 9 wt%), were prepared according to following procedure: The corresponding ionic liquid (IL) [BMIm][X] (X= Im, Cl) (6 g) was dissolved in distilled water (10 mL). The concentrated solution was mixture with 3 g of commercially available titania Evonix-P25 (TiO_2) under vigorous stirring for 30 minutes. The obtained solid was filtered and washed with distilled water (2 x 20 mL). The solid material was dried in vacuum for 3 h at 50 °C.

The others catalysts were prepared according to following procedure: The correspondent IL [BMIm][NTf_2], [BMPy][Im], [$(But)_3$EP][Im] (0.09 g) was added into a flask containing the TiO_2 (3 g). In the same flask 15 mL of $CHCl_3$ was add and the reaction was stirring for 30 minutes under ultrasound. After this time, the excess of solvent was removed by vacuum and the solid dried for 3 h at 50 °C.

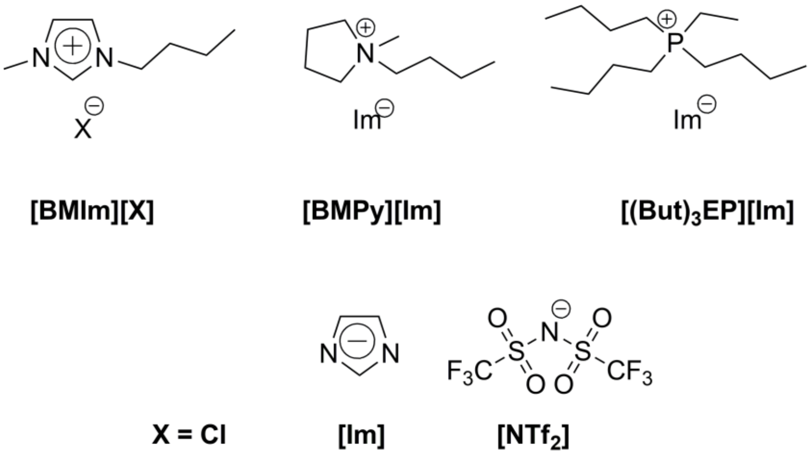

Figure S1. Structure and abbreviation of ILs used in this study.

2. NMR analysis of [BMIm][Im] IL before and after the photolysis

The NMR analyses were performed on a Bruker Avance 400 spectrometer, equipped with a BBO 5 mm probe with z-gradient operating at 400 MHz for ^1H, and 100 MHz for ^{13}C. The spectra were obtained at 298 K unless otherwise specified. Chemical shifts are reported in parts per million (ppm, δ) referenced to D_2O and DMSO-d_6 as an external reference (capillary).

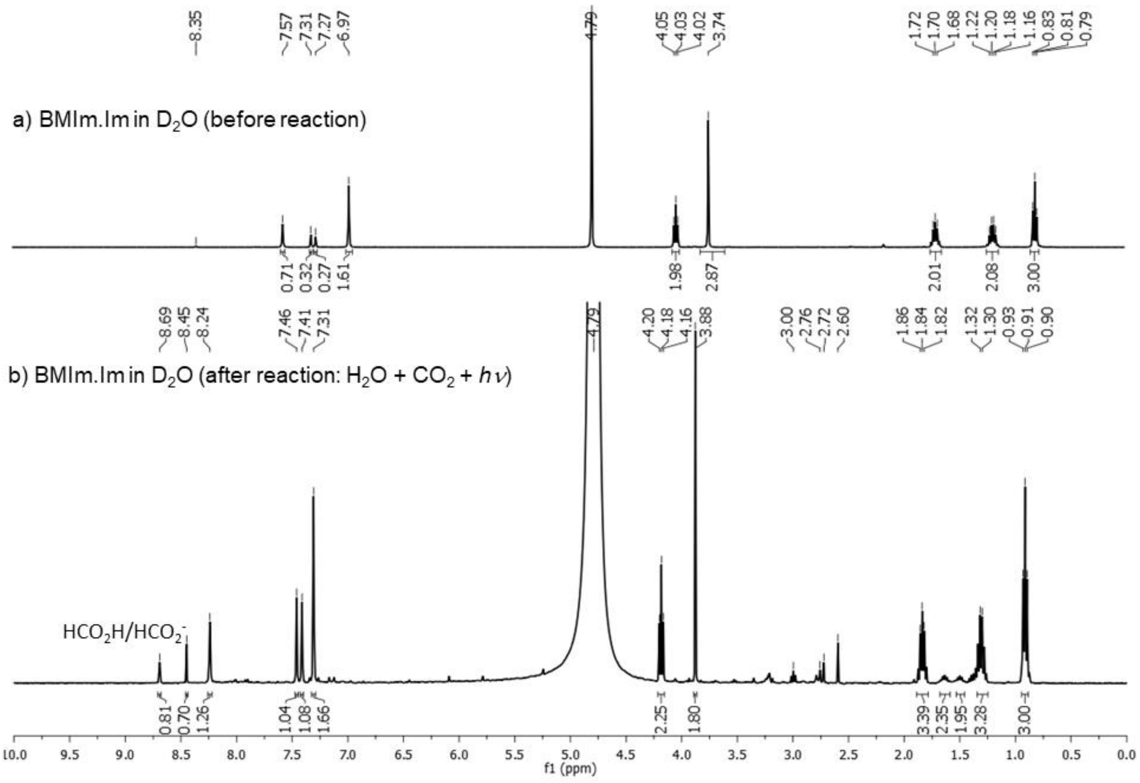

Figure S2. ^1H NMR (400 MHz, 25 °C) spectra of the aqueous solution of [BMIm][Im] IL: a) (in D_2O) before reaction; b) (DMSO-d_6 capillary) after reaction ($h\nu$ + CO_2).

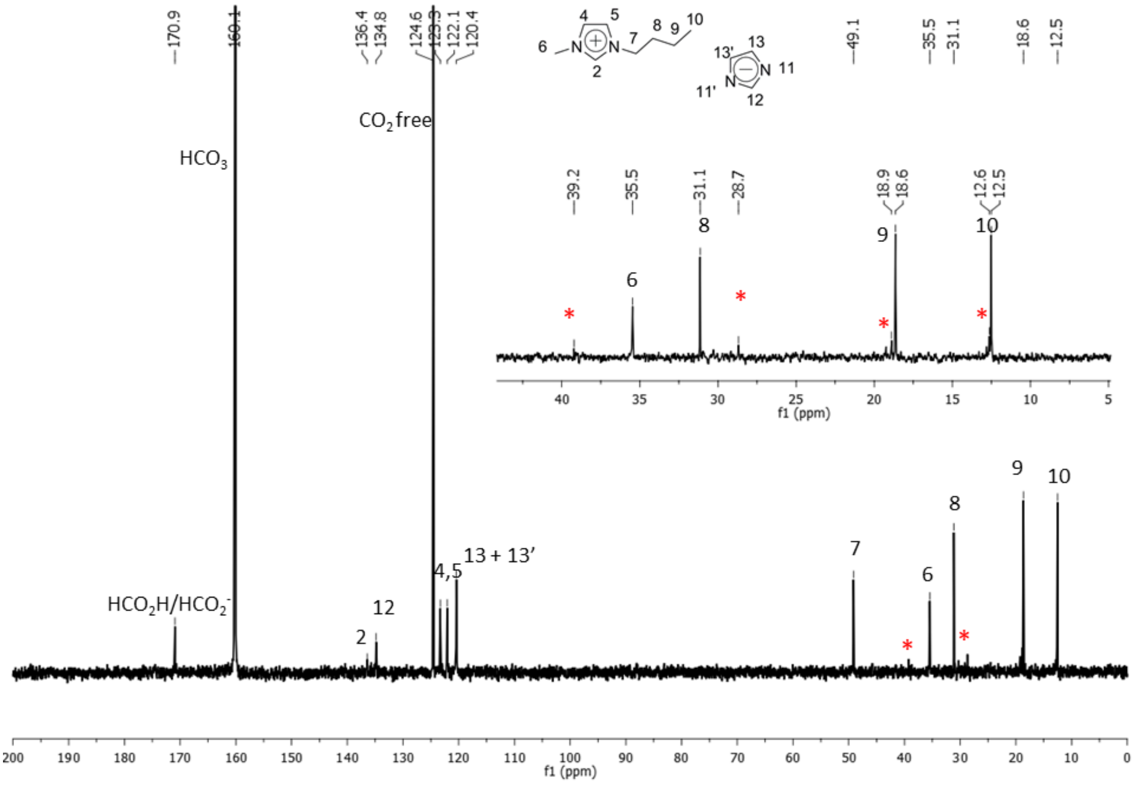

Figure S3. ^{13}C-{^{1}H} NMR (100 MHz, 25 °C) spectra of the aqueous solution of [BMIm][Im] IL (DMSO-d_6 capillary) after photoreaction ($h\nu$ + CO_2). No signal ~162 ppm of oxidized imidazolium ring (i.e 2-imidazolidinone) is observed, which exclude the possibilities that the unidentified products are the oxidized forms of the ionic liquid.

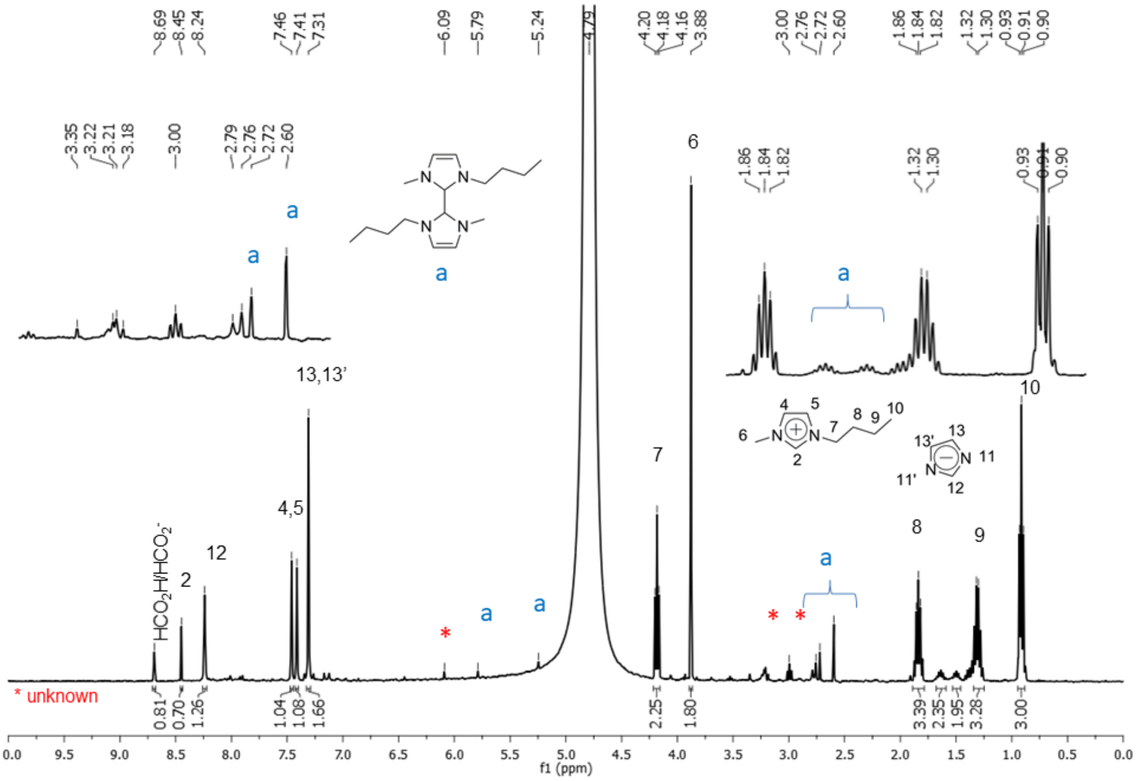

Figure S4. High zoom of ^{1}H NMR (400 MHz, 25 °C) spectra of aqueous solution of [BMIm][Im] IL (DMSO-d_6 capillary) after photoreaction ($h\nu$ + CO_2).

3. General Characterisation

Infra-Red (IR) analyses were conducted using a Bruker Alpha (Fourier transform infrared) FTIR spectrometer with an ATR attachment. Data collection utilised 256 cumulative scans with a resolution of 4 cm^{-1}. CHN Elemental analysis of the ILs immobilized on the support surfaces was carried out on a CHN Exeter Analytical CE-440 using helium (99.997% purity) as a carrier and oxygen for the combustion (99.995% purity). Thermogravimetric (TGA) analyses were performed in a TA Instruments T5500 Thermogravimetric analyser in a stepwise programmed up to 1000 ˚C (10 ˚C/min) using a nitrogen (99.995% purity) flow of 25 mL min^{-1}. Samples were held in Pt (high-temperature resistant) pans. Data analysis was processed using TRIOS Discovery program. N_2 isotherms of the catalysts, previously degassed at 180 °C under vacuum for 18 h, were obtained using TriStar and 3Flex Micromeritics instruments. Specific surface areas were determined by the BET multipoint method, and average pore size was obtained by the BJH method.

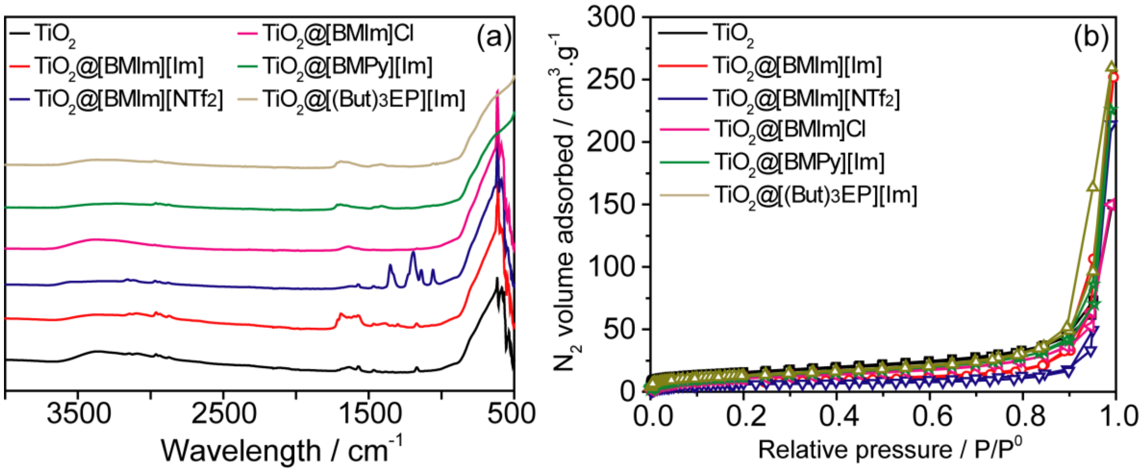

Figure S5. (a) FTIR absorbance and, (b) BET N_2-physisorption isotherms of pure TiO_2 and TiO_2 impregnated with [BMIm][Im], [BMIm][NTf2], [BMIm]Cl, [BMPy][Im] and [(But)3EP][Im].

Table S1. Textural properties of the TiO_2 and hybrid TiO_2@IL materials.

Entry	Photocatalyst	$Ratio_{C/N}$ [a]	IL[b] wt. %	S_{BET}[c] $m^2.g^{-1}$	V_{pore}[c] $cm^3.g^{-1}$
1	TiO_2	-	-	42.6[1]	0.017
2	TiO_2@[BMIm][Im]	2.8	3.2	19.2	0.007
3	TiO_2@[BMIm][NTf_2]	3.5	5.5	18.9	0.008
4	TiO_2@[BMIm]Cl	4.2	8.9	20.8	0.009
5	TiO_2@[BMPy][Im]	2.8	6.4	33.8	0.014
6	TiO_2@[$(But)_3$EP][Im]	7.2	6.5	29.2	0.012

[a]Determined by elemental analysis. [b]Determined by TGA analysis. [c]Determined by BET multipoint method and BJH method.

4. UV-Vis Diffuse Reflectance Spectroscopy

UV-Vis diffuse reflectance was performed by using a CARY 5000 spectrophotometer. TiO_2 shows an expected band gap of 3.3 eV[2] while the hybrid TiO_2@IL displayed a red shift of 0.2 eV independently of the IL.

5. Raman Spectroscopy

Raman spectroscopy was performed by using a Horiba-Jobin-Yvon LabRAM HR spectrometer, with a laser wavelength of 785 nm operating at a power of ca. 4 mW and a 600 lines mm^{-1} grating. Spectra were collected by averaging 2 acquisitions of 15 s duration. The presence of Anatase phase was observed for all samples and no significantly shift/change was observed in the peak position and FWHM (143.3 ± 0.1 cm^{-1} and 8.8 ± 0.1 cm^{-1}, respectively).[3] The higher intensity displayed for the samples with IL may suggest a decrease of the scattering due to the hybrid morphology (Table S1) which might lead an enhancement of the Raman signal. Indeed, deeper investigations are necessary to fully understand the cause of the Raman signal enhancement to hybrid TiO_2@IL, which will be done in the future since this is not the aim of this work.

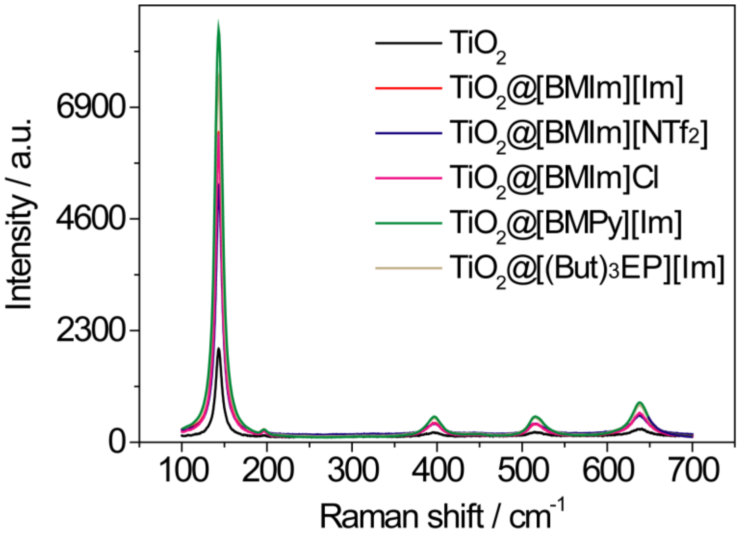

Figure S6. (a) Raman spectra of bare TiO_2 and TiO_2 impregnated with [BMIm][Im], [BMIm][NTf_2], [BMIm]Cl, [BMPy][Im] and [$(But)_3$EP][Im].

6. XPS Spectroscopy

XPS probes the upper surface of a material to depths of about 10 nm with XPS signal dominated by the surface of few nm. This allows the study of interface between TiO_2 and IL hybrid, and thus TiO_2@IL VB. Moreover, the area of analysed was ca. 0.5 mm^2 therefore probing TiO_2@IL average surface.

Powder sample were mounted on double sided tape (Sellotape) and pressed to ensure a good coverage of the tape with the powder. X-ray photoelectron spectroscopy (XPS) measurements were performed using a Kratos AXIS Ultra DLD instrument. The chamber pressure during the measurements was 5×10^{-9} Torr. Wide energy range survey scans were collected at pass energy of 80 eV in hybrid slot lens mode and a step size of 0.5 eV. Wide scan and high-resolution data on the C 1s, O 1s, N 1s, F 1s, S 2p, P 2p Ti 2p and VB photoelectron peaks was collected at pass energy 20 eV over energy ranges suitable for each peak, and collection times of 5 min, step sizes of 0.1 eV. The charge neutraliser filament was used to prevent the sample charging over the irradiated area. The X-ray source was a monochromated Al K_α emission, run at 10 mA and 12 kV (120 W). The energy range for each 'pass energy' (resolution) was calibrated using the Kratos Cu $2p_{3/2}$, Ag $3d_{5/2}$ and Au $4f_{7/2}$ three-point calibration method. The transmission function was calibrated using a clean gold sample method for all lens modes and the Kratos transmission generator software within Vision II. The data were processed with CASAXPS (Version 2.3.17). In XPS measurements an incorrect charge reference may induce misinterpretation of data, thus leading an equivocally explanation of the surface electronic structure of the materials. C 1s is the most used for charged reference XPS spectrum, however, for materials that contain a great amount of carbon in their composition (such as ionic liquids) the use of C 1s as reference is very uncertain. O 1s also can be used as charge reference, but can be very challenge to separate the contribution of the different oxidation states. Nonetheless, the C 1s and O

1s peaks are likely to contaminations such as adventitious carbon, water absorbed, etc. In this work, we have charged reference the XPS spectra using the Ti 2p at 458.9 eV[4] (Figure 7), therefore, all the shifts observed in the VBM are relative to Ti 2p. Ti 2p displayed a high number of counts/resolution and very narrow peak (around 1.2 eV for all the samples). Moreover, a non-significantly shift of Ti 2p signal for TiO_2-modified surface electronic structure was reported[5].

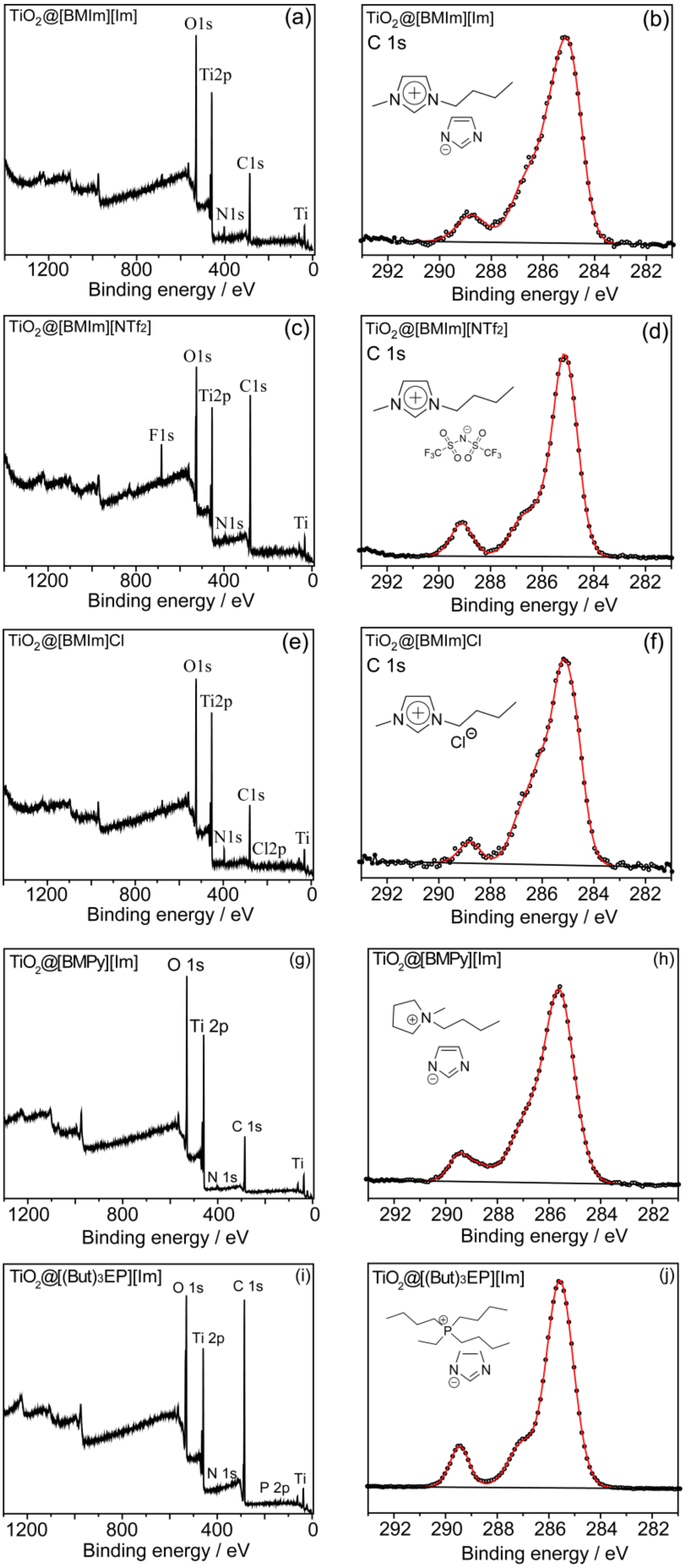

Figure S7. XPS wide scan and C 1s spectra of (a, b) TiO_2@[BMIm][Im]; (c, d) TiO_2@[BMIm][NTf_2]; (e, f) TiO_2@[BMIm]Cl; (g, h) [BMPy][Im] and (I, j) [$(But)_3$EP][Im].

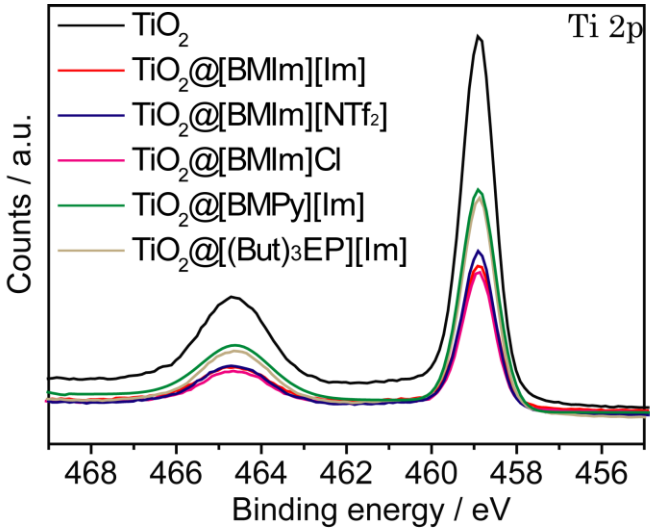

Figure S8. XPS-Ti 2p spectra of bare TiO_2 and TiO_2 impregnated with [BMIm][Im], [BMIm][NTf_2], [BMIm]Cl, [BMPy][Im] and [$(But)_3$EP][Im].

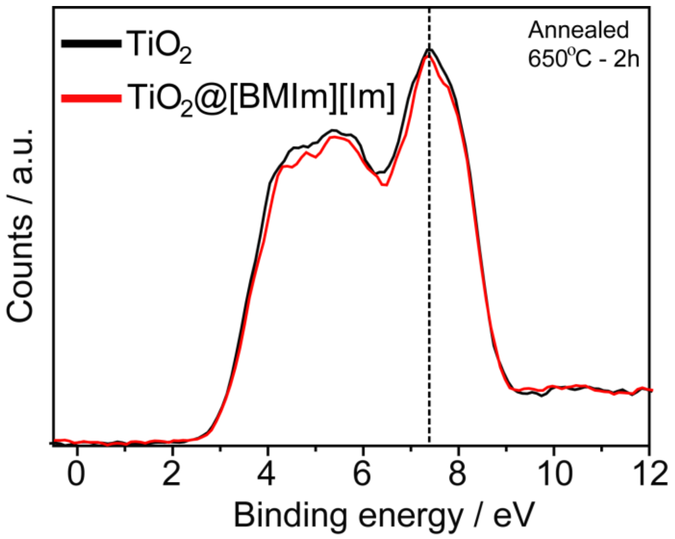

Figure S9. Valence band analysis of bare TiO_2 and TiO_2@[BMIm][Im] after annealing at 650ºC for 2 hours under air.

To ensure the appearance of new surfaces states found are ascribed only by the interaction between TiO_2 and IL, TiO_2@[BMIm][Im] was annealed at 650 °C for 2 h under air to remove the IL contribution from the TiO_2 surface. In addition, bare TiO_2 was also annealed at the same conditions, since at 650 °C crystalline structure rearrangement might induce changes in the VB. As can be observed in the Figure S10 the VBM of the samples annealed are located at same position (*ca.* 7.5 eV), which shows that the absence of IL on TiO_2 surface reverses it to its bare counterpart confirming our hypothesis.

7. Electrochemistry measurements

All the electrochemistry measurements were performed using TiO_2 and TiO_2@[BMIm][Im] films deposited on fluorine doped tin oxide (FTO) substrate, with each sample being prepared and measured three times to ensure the reproducibility of the measurements. Electrochemistry experiments were performed using an Autolab (PGSTAT 100N) potentiostat. The measurements were carried out in a quartz cell using a standard three-electrode configuration cell, with a platinum wire as the counter electrode, Ag/AgCl as the reference electrode and the photocatalyst as the working electrode; and 1 M NaOH media (pH 13.8). Mott Schottky measurements were performed in dark, in the frequency range of 100 kHz to 0.1 Hz at an amplitude of 10 mV.

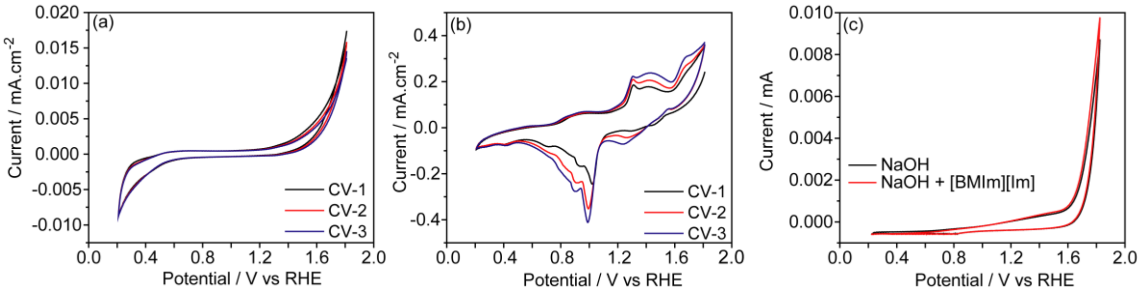

Figure S10. Cyclic voltammetry of **(a)** bare TiO_2 and **(b)** TiO_2@[BMIm][Im] using 1 M NaOH media, pH 14. **(c)** Cyclic voltammetries in 1 M NaOH and 0.1M of [BMIm][Im] in 1M NaOH were performed using Pt wires as working and counter electrodes. No additional peaks related to IL were observed in CVs; indicating IL possesses a wide electrochemical window. These results confirm the peaks observed for TiO_2@[BMIm][Im] in CV (Figure 2a) are related to the structural defects in TiO_2 surface due to presence of IL.

8. Photocatalytic CO_2 Reduction

Typically, a Schlenk tube containing 40 mL of degassed distilled water was saturated with 50 bar of CO_2 gas. 2 mL of Water was poured in a quartz reactor vessel containing 0.02 g of catalyst under argon atmosphere. A small vacuum was made in the reactor in order to remove air and argon. Then, CO_2 was inserted in the reactor by a balloon and stirred the reaction mixture for 30 min. Afterward, the balloon was removed and the reactor was placed in front of 300 W Xe lamp. The temperature of the reactor was maintained at 25ºC by circulated the cool water through its water jacket. After desired time, gaseous products were analysed by Agilent GC and the liquid phase was analysed by ^{1}H NMR.

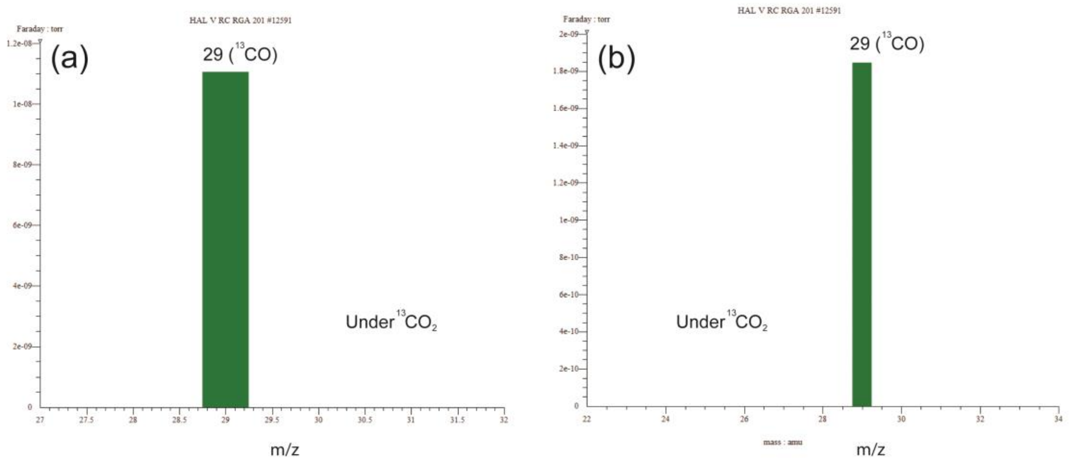

Figure S11. GC-MS spectra of ^{13}CO after photo-reduction of $^{13}CO_2$; (a) aqueous solution of [BMIm][Im] IL. Reaction conditions; [BMIm][Im] (40 mg), D_2O (2 mL), Temp. (25 °C), Time (1 h), Xe Lamp (300 W) and (b) TiO_2@[BMIm][Im] (30 mg), D_2O (2 mL), Temp. (25 °C), Time (1 h) and Xe Lamp (300 W). Ions detection was obtained by a Secondary Electron Multiplier (SEM) type detector, using the Single Ion Monitoring (SIM) method.

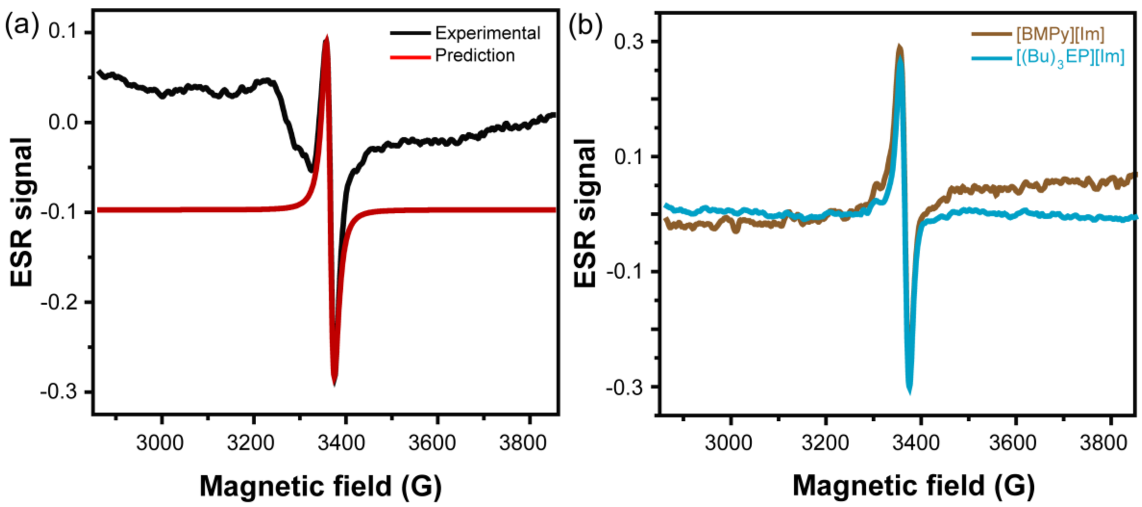

Figure S12. (a) Experimental and simulated ESR spectra of imidazole radical obtained from photoredduction of CO_2 in aqueous solution using of [BMIm][Im] IL; **(b)** imidazolale radical obtained photoredduction of CO_2 in aqueous solution using [BMPy][Im] and [(But)$_3$EP][Im] ILs.

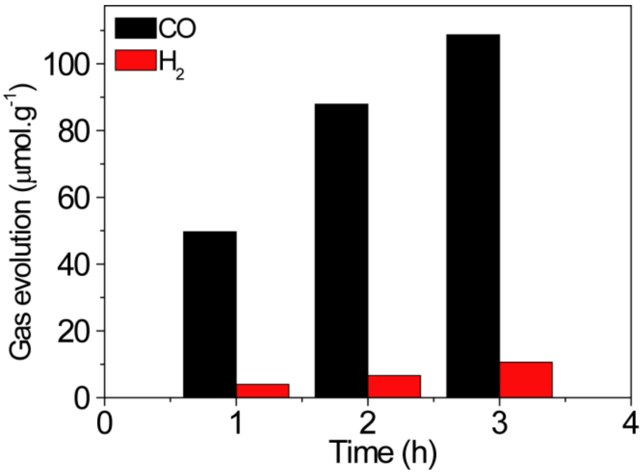

Figure S13. Photocatalysis using TiO_2@[BMIm][Im] in aqueous solution of formic acid (200 µl in 2 mL of H_2O), temp. (25 ˚C) and Xe lamp (300 W).

8. Recyclability tests

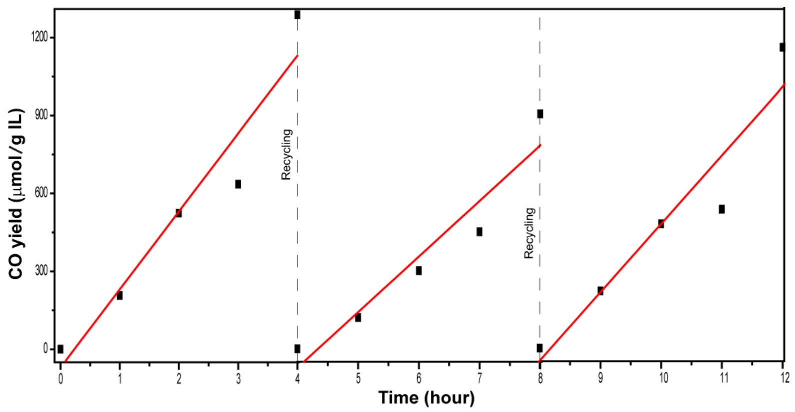

Figure S14. The recycling tests for CO_2 photo-reduction by TiO_2@[BMIm][Im] Reaction conditions: Cat. (20 mg, CO_2 (1 bar), H_2O (2 mL), temp. (25 ˚C) and Xe lamp (300 W). After each cycle the argon was passed to remove the CO and CO_2, then filled again the reactor with CO_2 and performed the reaction.

9. GC spectra of gaseous products

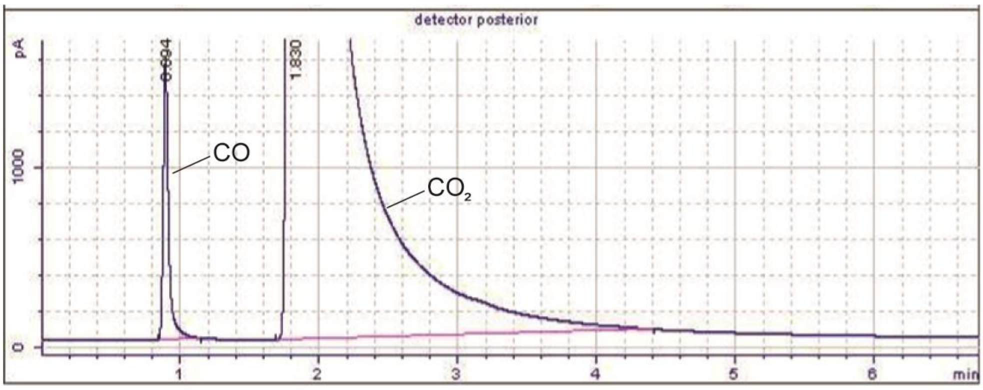

Figure S15. GC spectra of gaseous products after 2h reaction using TiO_2@[BMIm][Im].

Table S2. Recent examples of photocatalytic CO_2 conversions by TiO_2 based catalysts and ILs (main products).

Entry	Photocatalyst	Medium	T	Irradiation	CO (μmol.g^{-1}h^{-1})	CH_4	CH_3OH	C_{n+}	Ref.
			(K)			(μmol.g^{-1}h^{-1})			
**1**	**TiO_2@[BMIm][Im]**	**CO_2+H_2O**	**RT**	**240 W Xe arc lamp**	**455±96**	**-**	**-**	**-**	**This work**
2	5TC-OH/P25	CO_2+H_2O	RT	300 W Xe arc lamp	11.7	16.6	-	-	[6]
3	Pt/TiO_2	CO_2+H_2O	394	300 W Xe arc lamp	-	0.0081	-	-	[7]
4	Ru/TiO_2	CO_2+H_2O	394	300 W Xe arc lamp	-	0.0053	-	-	[7]
5	Au@STO/TiO_2	CO_2+H_2O	RT	300 W Xe arc lamp	160 ppm.cm^{-2}.h^{-1}	1100 ppm.cm^{-2}.h^{-1}	-	70 ppm.cm^{-2}.h^{-1}	[8]
6	Amine-TiO_2	CO_2+H_2O	RT	Xe lamp	100 ppm.h^{-1}	50 ppm.h^{-1}	-	-	[9]
7	Pt/TiO_2	CO_2+H_2 (2 bar CO_2)	323	100 W Xe arc lamp	1.1	5.2	-	-	[10]
8	CoAl/TiO_2 NTs	CO_2+H_2O	NF*	300 W Xe arc lamp	4.6	0.4	-	-	[11]
9	Ag/$BaLa4Ti_4O_{15}$	CO_2+H_2O	RT	400 W Hg arc lamp	73.3	-	-	-	[12]
10	TiO_2 (TiB(He)-Brookite)	CO_2+H_2O	303	90 mW/cm^2 AM 1.5G	3	3.2	-	-	[13]
11	5TC-OH/TiO_2	CO_2+H_2O	RT	300 W Xe lamp	11.7	16.6	-	-	[14]
12	OMT-Au	CO_2+H_2O	NF*	300 W Xe arc lamp	1.8	0.4	-	-	[15]
13	Pd_7Cu_1-TiO_2	CO_2+H_2O (0.2 MPa)	NF*	2-mWcm^{-2} - cut-off filter 400 nm	1.9	19.6	-	-	[16]
14	Pt-TiO_2	CO_2+H_2O	303	Xe lamp, 19.6 mW/cm^2	200.0 μmol/g-cat/h	1361.0 μmol/g-cat/h	-	-	[17]
15	$TiO2_{2-x}$ 001-101	CO_2+H_2O	423	100 W mercury vapor lamp	11.0	-	-	-	[18]
16	TiO_2 (HF4.5)	CO_2+H_2O+4M HCl	NF*	300 W Xe arc lamp	-	1.4	-	-	[19]
17	(Au, Cu)/TiO_2 (Au/Cu 1:2)	CO_2+H_2O (1.7 atm)	333	1000 mWcm^{-2} - filter AM1.5	-	2200.0 μmol/g-cat/h	-	-	[20]
18	G2-TiO_2	CO_2+H_2O	NF*	300 W Xe arc lamp	-	8.0	-	16.8	[21]

19	0.5% $Ni(OH)_2$ -TiO_2	CO_2+H_2O	NF*	Xe lamp, 40 mW/cm^2	0.7	2.2	0.1	0.2	[22]
20	Au@CdS/IO-TiO_2-1	CO_2+H_2O	RT	Xe lamp, 100 mW/cm^2	0.6	41.6	-	-	[23]
21	(TiO_2)-Cu/BTN	CO_2+H_2O	NF*	300 W Xe arc lamp	5.0	17.5	-	-	[24]
22	CuPt-TiO_2	CO_2+H_2O (1.2 atm)	313	150 W Xe arc lamp	-	11.3	-	-	[25]
23	Au-Ru/TiO_2	CO_2+H_2	323	Hg lamp, 150 mW/cm^2	-	82.3	-	11.4	[26]
24	(G-$Ti_{0.91}O_2$)$_5$	CO_2+H_2O	NF*	300 W Xe arc lamp	8.9	1.2	-	-	[27]
25	(OMT-2) ordered mesoporous TiO_2	CO_2+H_2O (80 kPa)	NF*	300 W Xe arc lamp	0.15	0.2	-	-	[28]
26	Cu/TiO_2	CO_2+H_2O (0.2 MPa)	323	200 W Xe arc lamp (λ = 320-780 nm)	5.4	8.7	-	-	[29]
27	EMIM.BF_4, H_2O, CO_2, $[Ru(bpy)_3]Cl_2$, $CoCl_2.6H_2O$, TEOA		303	300 W Xe arc lamp - cut-off filter 420 nm	15.5 □mol/h	-	-	-	[30]

* Not found

Table S3. Recent examples of photocatalytic CO_2 conversions (main products).

Entry	Photocatalyst	Medium	T (K)	Irradiation	CO	CH_4 (μmol.g^{-1}h^{-1})	CH_3OH	C_{n+}	Ref.
1	Ru/Al_2O_3	CO_2+H_2	394	300 W Xe arc lamp	-	18.2 x 10^6	trace	-	[7]
2	Ru/$NaTaO_3$	CO_2+H_2O	394	300 W UV-enhanced Xe	trace	51.8	-	-	[31]
3	Pt/$NaTaO_3$	CO_2+H_2O	RT	300 W UV-enhanced Xe	139.1	trace	-	-	[31]
4	Ag/$BaZrO_3$	CO_2+H_2O	RT	300 W Xe lamp	-	0.9	-	-	[32]
5	IL-Conjugated Polymer	CO_2+H_2O+TEOA	RT	Visible light	47.4	-	-	-	[33]
6	Ni(TPA/TEG)	$[Ru(bpy)_3]Cl_2·6H_2O$ + H_2O/acetonitrile + TEOA + CO_2	NF*	300 W Xe lamp – visible light	1.6 x 10^4	-	-	-	[34]
7	g-C_3N_4/ZnO	CO_2+H_2O (vapor)	RT	300 W Xe arc lamp	-	-	0.6	-	[35]
8	meso-CdSe	CO_2+H_2O	RT	300 W Xe arc lamp – cut-off filter 420 nm	6.0	0.5	-	-	[36]
9	Meso-ZnS	CO_2+H_2O	RT	300 W Xe arc lamp – cut-off filter 420 nm	1.8	3.8	-	-	[36]
10	NC@Co_2O_4	$[Ru(bpy)_3]Cl_2·6H_2O$ + H_2O/acetonitrile + TEOA + CO_2	303	300 W Xe arc lamp – cut-off filter 420 nm	2.6 x 10^4	-	-	-	[37]
11	α-Fe_2O_3/g-C_3N_4	CO_2+H_2O	RT	Xe lamp with a focus intensity of 0.21 W cm^{-2}	27.2	-	-	-	[38]
12	15CN-6Al-F (15CN-6Al-F)	CO_2+H_2O	NF*	300 W Xe lamp	24.0	6.0	-	-	[39]
13	In_2S_3-$CdIn_2S_4$ NTs	H_2O+CO_2+ACN+Co(bpy)$_3^{2+}$ (bpy=2'2-bipyridine)+TEOA	303	Cut-off filter 400 nm	825.0	-	-	-	[40]

14	O-doped g-C_3N_4	CO_2+H_2O	NF*	350 W Xe lamp - cut-off filter 420 nm	-	-	0.88	-	[41]
15	Ag/Yb-modified Ga_2O_3	0.1M $NaHCO_3$	NF*	400 W high-pressure mercury lamp	4.5 mmol of CO after 39 h photoirradiation				[42]
16	Co_1–G nanosheets	[Ru(bpy)3] CO_2+Cl_2·6H_2O (bpy = 2′2-bipyridine)+H_2O/ triethanolamine/ACN	15	264.25 mW cm^{-2} - cut-off filter 420 nm	CO : TON of 350 in the first 3 h				[43]
17	$LaCoO_3$ perovskite	CO_2+Ru(bpy)$_3$$Cl_2$·6 H_2O+TEOA+ MeCN : H_2O	303	300 W Xe arc lamp – cut-off filter 420 nm	44.2 µmol.h^{-1} of CO				[44]
18	BOC-OV	CO_2+H_2O	RT	300 W high pressure xenon lamp	17.0	1.0	-	-	[45]
19	[EMIM][BF_4], H_2O, CO2, [Ru(bpy)$_3$]Cl_2, $CoCl_2$.6H_2O, TEOA		303	300 W Xe arc lamp – cut-off filter 420 nm	31.0	-	-	-	[30]
20	g-C_3N_4 nanosheets	CO_2+H_2O	NF*	300 W Xe arc lamp	-	1.4	1.9	-	[46]
21	CeO_2 homojunction (CP2)	CO_2+H_2O	NF*	300 W Xe arc lamp	-	0.86	-	-	[47]
22	Mg-In LDH	CO_2+H_2O	NF*	200 W Hg-Xe arc lamp	3.2	-	-	-	[48]
23	MOF-525-Co	CO_2+ MeCN:TEOA (4:1) – (80kPa)	NF*	300 W Xe arc lamp – 400 nm <λ< 800 nm	200.6	36.8	-	-	[49]

* Not found

References

[1] aJ. S. Kang, J. Lim, W.-Y. Rho, J. Kim, D.-S. Moon, J. Jeong, D. Jung, J.-W. Choi, J.-K. Lee, Y.-E. Sung, *Scientific Reports* **2016**, *6*, 30829; bB. O'Regan, F. Lenzmann, R. Muis, J. Wienke, *Chemistry of Materials* **2002**, *14*, 5023-5029.

[2] aJ. A. Fernandes, E. C. Kohlrausch, S. Khan, R. C. Brito, G. J. Machado, S. R. Teixeira, J. Dupont, M. J. Leite Santos, *Journal of Solid State Chemistry* **2017**, *251*, 217-223; bJ. A. Fernandes, P. Migowski, Z. Fabrim, A. F. Feil, G. Rosa, S. Khan, G. J. Machado, P. F. P. Fichtner, S. R. Teixeira, M. J. L. Santos, J. Dupont, *Physical Chemistry Chemical Physics* **2014**, *16*, 9148-9153.

[3] A. Li Bassi, D. Cattaneo, V. Russo, C. E. Bottani, E. Barborini, T. Mazza, P. Piseri, P. Milani, F. O. Ernst, K. Wegner, S. E. Pratsinis, *Journal of Applied Physics* **2005**, *98*, 074305.

[4] aX. Han, K. Song, L. Lu, Q. Deng, X. Xia, G. Shao, *Journal of Materials Chemistry C* **2013**, *1*, 3736-3746; bT. Leichtweiss, R. A. Henning, J. Koettgen, R. M. Schmidt, B. Hollander, M. Martin, M. Wuttig, J. Janek, *Journal of Materials Chemistry A* **2014**, *2*, 6631-6640.

[5] J. Tao, T. Luttrell, M. Batzill, *Nature Chemistry* **2011**, *3*, 296.

[6] M. Ye, X. Wang, E. Liu, J. Ye, D. Wang, *ChemSusChem*, n/a-n/a.

[7] X. Meng, T. Wang, L. Liu, S. Ouyang, P. Li, H. Hu, T. Kako, H. Iwai, A. Tanaka, J. Ye, *Angewandte Chemie International Edition* **2014**, *53*, 11478-11482.

[8] Q. Kang, T. Wang, P. Li, L. Liu, K. Chang, M. Li, J. Ye, *Angewandte Chemie International Edition* **2015**, *54*, 841-845.

[9] Y. Liao, S.-W. Cao, Y. Yuan, Q. Gu, Z. Zhang, C. Xue, *Chemistry – A European Journal* **2014**, *20*, 10220-10222.

[10] S. Xie, Y. Wang, Q. Zhang, W. Deng, Y. Wang, *ACS Catalysis* **2014**, *4*, 3644-3653.

[11] S. Kumar, L. J. Durndell, J. C. Manayil, M. A. Isaacs, C. M. A. Parlett, S. Karthikeyan, R. E. Douthwaite, B. Coulson, K. Wilson, A. F. Lee, *Particle & Particle Systems Characterization* **2018**, *35*, 1700317-n/a.

[12] K. Iizuka, T. Wato, Y. Miseki, K. Saito, A. Kudo, *Journal of the American Chemical Society* **2011**, *133*, 20863-20868.

[13] L. Liu, H. Zhao, J. M. Andino, Y. Li, *ACS Catalysis* **2012**, *2*, 1817-1828.

[14] M. Ye, X. Wang, E. Liu, J. Ye, D. Wang, *ChemSusChem* **2018**, doi:10.1002/cssc.201800083.

[15] H. Xue, T. Wang, H. Gong, H. Guo, X. Fan, B. Gao, Y. Feng, X. Meng, X. Huang, J. He, *Chemistry – An Asian Journal*, n/a-n/a.

[16] R. Long, Y. Li, Y. Liu, S. Chen, X. Zheng, C. Gao, C. He, N. Chen, Z. Qi, L. Song, J. Jiang, J. Zhu, Y. Xiong, *Journal of the American Chemical Society* **2017**, *139*, 4486-4492.

[17] W.-N. Wang, W.-J. An, B. Ramalingam, S. Mukherjee, D. M. Niedzwiedzki, S. Gangopadhyay, P. Biswas, *Journal of the American Chemical Society* **2012**, *134*, 11276-11281.

[18] L. Liu, Y. Jiang, H. Zhao, J. Chen, J. Cheng, K. Yang, Y. Li, *ACS Catalysis* **2016**, *6*, 1097-1108.

[19] J. Yu, J. Low, W. Xiao, P. Zhou, M. Jaroniec, *Journal of the American Chemical Society* **2014**, *136*, 8839-8842.

[20] Ş. Neaţu, J. A. Maciá-Agulló, P. Concepción, H. Garcia, *Journal of the American Chemical Society* **2014**, *136*, 15969-15976.

[21] W. Tu, Y. Zhou, Q. Liu, S. Yan, S. Bao, X. Wang, M. Xiao, Z. Zou, *Advanced Functional Materials* **2013**, *23*, 1743-1749.

[22] a. meng, s. wu, b. cheng, J. Yu, J. Xu, *Journal of Materials Chemistry A* **2018**.

[23] Y. Wei, J. Jiao, Z. Zhao, J. Liu, J. Li, G. Jiang, Y. Wang, A. Duan, *Applied Catalysis B: Environmental* **2015**, *179*, 422-432.

[24] J. Jin, J. Luo, L. Zan, T. Peng, *ChemPhysChem* **2017**, *18*, 3230-3239.

[25] S. Lee, S. Jeong, W. D. Kim, S. Lee, K. Lee, W. K. Bae, J. H. Moon, S. Lee, D. C. Lee, *Nanoscale* **2016**, *8*, 10043-10048.

[26] L. Zhang, G. Kong, Y. Meng, J. Tian, L. Zhang, S. Wan, J. Lin, Y. Wang, *ChemSusChem* **2017**, *10*, 4709-4714.

[27] W. Tu, Y. Zhou, Q. Liu, Z. Tian, J. Gao, X. Chen, H. Zhang, J. Liu, Z. Zou, *Advanced Functional Materials* **2012**, *22*, 1215-1221.

[28] T. Wang, X. Meng, P. Li, S. Ouyang, K. Chang, G. Liu, Z. Mei, J. Ye, *Nano Energy* **2014**, *9*, 50-60.

[29] Q. Zhai, S. Xie, W. Fan, Q. Zhang, Y. Wang, W. Deng, Y. Wang, *Angewandte Chemie International Edition* **2013**, *52*, 5776-5779.
[30] J. Lin, Z. Ding, Y. Hou, X. Wang, *Scientific Reports* **2013**, *3*, 1056.
[31] M. Li, P. Li, K. Chang, T. Wang, L. Liu, Q. Kang, S. Ouyang, J. Ye, *Chemical Communications* **2015**, *51*, 7645-7648.
[32] X. Chen, J. Wang, C. Huang, S. Zhang, H. Zhang, Z. Li, Z. Zou, *Catalysis Science & Technology* **2015**, *5*, 1758-1763.
[33] Y. Chen, G. Ji, S. Guo, B. Yu, Y. Zhao, Y. Wu, H. Zhang, Z. Liu, B. Han, Z. Liu, *Green Chemistry* **2017**, *19*, 5777-5781.
[34] K. Niu, Y. Xu, H. Wang, R. Ye, H. L. Xin, F. Lin, C. Tian, Y. Lum, K. C. Bustillo, M. M. Doeff, M. T. M. Koper, J. Ager, R. Xu, H. Zheng, *Science Advances* **2017**, *3*.
[35] W. Yu, D. Xu, T. Peng, *Journal of Materials Chemistry A* **2015**, *3*, 19936-19947.
[36] Y. Y. Lee, H. S. Jung, J. M. Kim, Y. T. Kang, *Applied Catalysis B: Environmental* **2018**, *224*, 594-601.
[37] S. Wang, B. Y. Guan, X. W. Lou, *Energy & Environmental Science* **2018**, *11*, 306-310.
[38] Z. Jiang, W. Wan, H. Li, S. Yuan, H. Zhao, P. K. Wong, *Advanced Materials*, 1706108-n/a.
[39] J. Wang, C. Qin, H. Wang, M. Chu, A. Zada, X. Zhang, J. Li, F. Raziq, Y. Qu, L. Jing, *Applied Catalysis B: Environmental* **2018**, *221*, 459-466.
[40] S. Wang, B. Y. Guan, Y. Lu, X. W. D. Lou, *Journal of the American Chemical Society* **2017**, *139*, 17305-17308.
[41] J. Fu, B. Zhu, C. Jiang, B. Cheng, W. You, J. Yu, *Small* **2017**, *13*, 1603938-n/a.
[42] H. Tatsumi, K. Teramura, Z. Huang, Z. Wang, H. Asakura, S. Hosokawa, T. Tanaka, *Langmuir* **2017**, *33*, 13929-13935.
[43] C. Gao, S. Chen, Y. Wang, J. Wang, X. Zheng, J. Zhu, L. Song, W. Zhang, Y. Xiong, *Advanced Materials*, 1704624-n/a.
[44] J. Qin, L. Lin, X. Wang, *Chemical Communications* **2018**.
[45] Z. Ma, P. Li, L. Ye, Y. Zhou, F. Su, C. Ding, H. Xie, Y. Bai, P. K. Wong, *Journal of Materials Chemistry A* **2017**, *5*, 24995-25004.
[46] P. Xia, B. Zhu, J. Yu, S. Cao, M. Jaroniec, *Journal of Materials Chemistry A* **2017**, *5*, 3230-3238.
[47] P. Li, Y. Zhou, Z. Zhao, Q. Xu, X. Wang, M. Xiao, Z. Zou, *Journal of the American Chemical Society* **2015**, *137*, 9547-9550.
[48] K. Teramura, S. Iguchi, Y. Mizuno, T. Shishido, T. Tanaka, *Angewandte Chemie International Edition* **2012**, *51*, 8008-8011.
[49] H. Zhang, J. Wei, J. Dong, G. Liu, L. Shi, P. An, G. Zhao, J. Kong, X. Wang, X. Meng, J. Zhang, J. Ye, *Angewandte Chemie International Edition* **2016**, *55*, 14310-14314.
